# Diagnostic Dilemma in a Septuagenarian Patient: Drug-Induced Liver Injury (DILI) or Autoimmune Hepatitis?

**DOI:** 10.7759/cureus.84253

**Published:** 2025-05-16

**Authors:** Chibueze Nnonyelu

**Affiliations:** 1 Internal Medicine, SSM Health St. Joseph Hospital, Saint Charles, USA

**Keywords:** autoimmune hepatitis (aih), diagnosis, drug-induced liver injury (dili), elderly people, inflammatory disease, non-alcoholic steatohepatitis (nash)

## Abstract

Autoimmune hepatitis is an uncommon, long-term condition in which the immune system targets the liver, leading to inflammation. It can affect individuals of any age or background and often presents either without symptoms or with vague complaints such as tiredness, appetite loss, nausea, or weight reduction. While it can emerge at any point in life, it tends to occur more frequently during adolescence and again in later adulthood. This report describes the case of an elderly woman of Indian origin who was found to have elevated liver enzymes with no clear cause. Subsequent evaluations, including liver biopsy, confirmed autoimmune hepatitis. This case highlights the steps taken to reach a diagnosis and the complexities involved in choosing a suitable management plan.

## Introduction

Autoimmune hepatitis (AIH) is an inflammatory disease of the liver of unknown cause that may progress to liver cirrhosis and end-stage liver failure if diagnosis is overlooked and treatment is delayed [1]. It is a rare chronic liver disease with a variable clinical presentation. A systematic review and meta-analysis found the global incidence and prevalence to be 1.37 (95%CI: 0.95-1.80) and 17.44 (95%CI: 12.01-22.87) per 100,000 persons, respectively [2]. It has a bimodal distribution, with a peak in the second decade and another peak between the fifth and sixth decades [3]. It is rarely diagnosed in the elderly, requiring a high index of suspicion and ruling out other mimics or differentials.

## Case presentation

A 72-year-old female patient of Indian descent with a past medical history of hypertension, hyperlipidemia, and osteoporosis presented with two weeks of generalized weakness and anorexia. She reported vague upper abdominal pain, bloating, weight loss, and itchiness. Outpatient blood work revealed elevated liver enzymes and hyperbilirubinemia, which were normal six months ago. She did not drink alcohol. She reported traveling to India six months prior, where she admitted taking some herbal medications, the names of which and duration of intake could not be ascertained. She denied the use of Tylenol, non-steroidal anti-inflammatory drugs (NSAIDs), or steroids. Physical exams showed BMI of 25kg/m^2^. Laboratory test results are included in Table 1.

**Table 1:** Lab findings AST: aspartate transaminase; ALT: alanine transaminase; ALP: alkaline phosphatase; IgG: immunoglobin G; Na: sodium

Parameter and unit	Patient value	Reference range
Na (mmol/l)	130	135-145
ALP (U/L)	1167	40-150
ALT (U/L)	628	5-55
AST (U/L)	1214	5-34
Total Bilirubin (mg/dl)	6.7	0.2-1.2
Direct Bilirubin (mg/dl)	4	0.10-0.50
Hemoglobin (g/dl)	11.1	11.9-15.8
IgG (mg/dl)	1995	767-1590

Other labs included C-reactive protein (CRP) 0.66mg/dl (<0.5), INR 1.1, anti-nuclear antibodies (ANA) negative, anti-smooth muscle antibody (ASMA) positive titer 1: 320, anti-mitochondrial antibodies (AMA) <20, hepatitis A virus non-reactive, hepatitis B antigen negative, hepatitis C antibody negative, anti-liver kidney microsome (LKM) antibody negative, anti-soluble liver antibody negative, and anti-liver cytosol antibody negative. 

**Figure 1:**
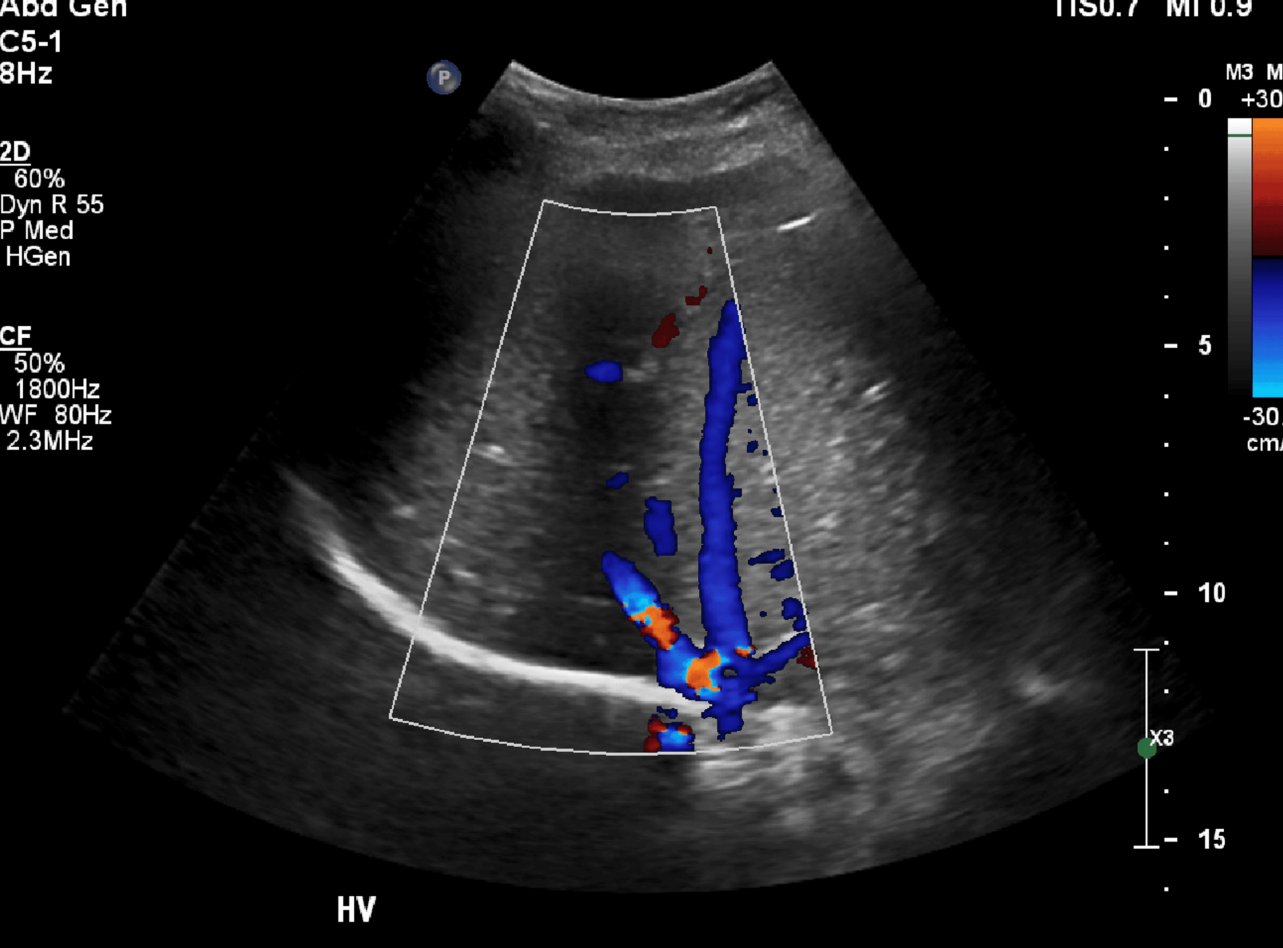
Doppler ultrasound of the liver showing patent portal vein with no filling defect

**Figure 2:**
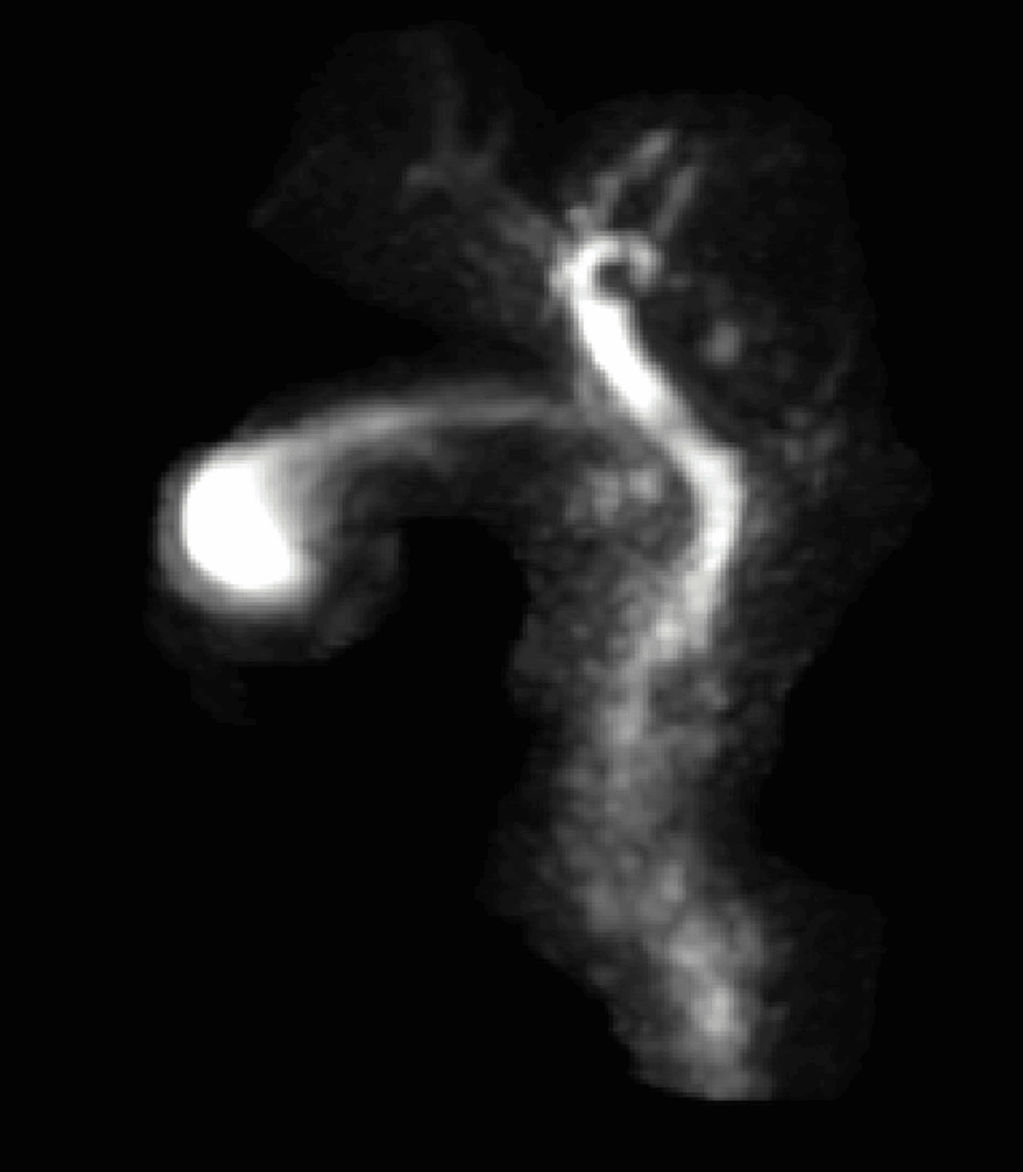
Magnetic resonance cholangiopancreatography showing no biliary dilatation or filing defects

CT abdomen/pelvis was negative for hepatomegaly or fatty infiltration. Doppler color liver ultrasonography (Figure 1) showed patent hepatic vein with no filling defect. MRI/magnetic resonance cholangiopancreatography (MRCP) (Figure 2) showed no biliary or pancreatic dilatation or filling defect. Liver biopsy showed chronic hepatitis with mild portal lymphoplasmacytic infiltration, interface hepatitis with biliary plugs. There were no iron deposits, no portal fibrosis. Diagnosis of AIH was made following a review of laboratory findings and then confirmed with a liver biopsy.

Treatment ideally includes steroids ± azathioprine or 6-mercaptopurine. However, considering the patient's age and history of osteoporosis, treatment was deferred. She was monitored closely and was asked to avoid hepatotoxic agents like Tylenol and contrast. On the three-week follow-up, alkaline phosphatase (ALP) was 725 U/L, aspartate transaminase (AST) was 162 U/L, alanine transaminase (ALT) was 146 U/L, and total bilirubin was 1 mg/dl.

## Discussion

AIH is a rare chronic disease with an estimated prevalence of approximately 10-50/100,000 in the Western population, with a strong predominance in middle-aged women [4]. A systematic retrospective analysis done by Thawab et al. to evaluate AIH in the elderly showed that elderly patients more frequently present with severe disease [4].

It has a broad range of clinical features, ranging from asymptomatic to extremely debilitating symptoms, including fatigue, anorexia, and weight loss. Workup involves biochemical tests and autoantibody screening. In acute cases, AST and ALT levels may exceed 10-20 times the upper limit of normal, and the ratio of ALP to AST (or ALT) is often less than 1:5, and in some cases, less than 1:10 [5]. In chronic presentations, AST and ALT levels are typically 1.5-5 times the upper limit of normal, with the ALP to AST or ALT ratio averaging around 1:2 [5]. The autoantibodies associated with AIH include ANA with titers of 1:80 to 1:100, and ASMA, which are more specific. Other relevant antibodies include anti-soluble liver antigen/liver pancreas antibody (anti-SLA/LP) and anti-LKM-1, which are primarily seen in type 2 AIH [6]. AMA, although typically associated with primary biliary cholangitis, may be present in overlap syndromes involving AIH [7].

Diagnosis is made with a simplified scoring system: ANA or SMA ≥1:40 (1 point), ANA or SMA ≥1:80 or LKM1 ≥1:40 or SLA positive (2 points), serum IgG upper limit of normal (1 point), serum IgG >1.1 x upper limit of normal (2 points), histology compatible (presence of interface hepatitis but not all three features are seen) with AIH (2 points), histology typical (presence of all three major features: interface hepatitis, rosettes, and emperipolesis) of AIH (2 points), negative hepatitis viral markers (2 points). Probable diagnosis is made if the total points are 6, and a definite diagnosis is made when points are ≥ 7 [8]. This is then confirmed with a liver biopsy. 

AIH is classified into Type I and Type II based on serological, clinical, and genetic parameters. Type 1 AIH has positive ANA and SMA, while Type II has positive anti-LKM antibodies [3].

Imaging such as CT abdomen is routinely not required to make the diagnosis; however, it could detect fatty infiltration of the liver as seen in hepatic steatohepatitis, which is a close differential. MRI/MRCP is useful in patients with cholestasis to exclude possible primary sclerosing cholangitis [9]. Liver biopsy is critical for diagnosis and typically shows interface hepatitis with portal mononuclear cell infiltrate (generally lymphoplasmacytic, often with occasional eosinophils), which spares the biliary tree [10]. Differentiating drug-induced liver injury (DILI) from AIH histologically is challenging; however, the presence of portal neutrophils leans toward DILI.

Treatment involves steroids ± azathioprine or 6-mercaptopurine. The initiation of treatment usually involves evaluating risks and benefits as well as quality of life. In elderly patients with comorbidities, conservative management may be preferred. The patient in the current report demonstrated improvement in liver enzymes over three weeks without treatment, supporting a watchful-waiting approach.

## Conclusions

AIH is rare in the elderly and may be mistaken for other hepatic conditions, including DILI. Accurate diagnosis requires comprehensive evaluation, including serology and liver biopsy. In certain elderly patients with contraindications to immunosuppressants, close monitoring may be a viable alternative to immediate treatment.
